# Transitioning from invasive to liquid biopsy techniques: a bibliometric analysis and prospective insights on biomarkers in lupus nephritis

**DOI:** 10.3389/fmed.2026.1796760

**Published:** 2026-05-11

**Authors:** Chen Liu, Yanhao Chen, Xiang Li, Shuo Huang, Yongsheng Fan, Jie Bao

**Affiliations:** 1 The Second School of Clinical Medicine, Zhejiang Chinese Medical University, Hangzhou, Zhejiang, China; 2 School of Basic Medical Sciences, Zhejiang Chinese Medical University, Hangzhou, Zhejiang, China

**Keywords:** bibliometric analysis, biomarkers, liquid biopsy, lupus nephritis, visual analysis

## Abstract

**Background:**

Lupus nephritis (LN) significantly contributes to morbidity and mortality in systemic lupus erythematosus (SLE) patients. Although renal biopsy is the diagnostic “gold standard,” its invasiveness limits ongoing disease monitoring. Recently, research has focused on non-invasive “liquid biopsy” biomarkers, especially in urine. This study uses bibliometric methods to analyze the development and trends in LN biomarker research, highlighting recent advancements and future directions.

**Methods:**

We collected original research articles on LN biomarkers from the Web of Science Core Collection and PubMed databases from 2005 to 2024. We conducted a bibliometric analysis using CiteSpace, VOSviewer, and Bibliometrix, and included 2025 studies on urinary biomarkers, renal pathology, disease activity, and long-term outcomes.

**Results:**

The analysis encompassed 576 articles, revealing three distinct developmental phases in the field, accompanied by a notable surge in publications since 2016. China and the United States are the top contributors, with the U.S. leading in citation impact and collaboration. The most active journal functions as a crucial repository of knowledge. Keyword and citation analysis reveal a shift from traditional immunological markers to non-invasive urinary biomarkers, such as MCP-1, NGAL, CD163, and complement-related molecules, alongside the adoption of multi-omics approaches and predictive models.

**Conclusion:**

Research on LN biomarkers is moving from discovery to clinical application. Advances in single-cell and spatial omics are shifting focus from peripheral biomarkers to renal molecular phenotypes, supporting “liquid biopsy” strategies. Future efforts will emphasize AI-driven multi-omics integration, minimal biomarker panels, and cross-ethnic validation to develop a precise, non-invasive LN monitoring system.

## 1 Introduction

Lupus nephritis (LN) represents the most severe and prevalent form of organ involvement in systemic lupus erythematosus (SLE), with renal impairment manifesting in approximately 60% of individuals diagnosed with SLE as the disease progresses (1). Even with improvements in immunosuppressive treatments that have enhanced patient outcomes, 5–20% of patients still advance to end-stage renal disease (ESRD) within 15 years of being diagnosed. This advancement greatly raises the risk of death and places a substantial financial strain on healthcare systems (2, 3). The pathological mechanisms underlying LN are intricate, characterized by the deposition of immune complexes, excessive activation of the complement system, and subsequent inflammatory cascades, which ultimately result in irreversible damage to glomerular and tubulointerstitial structures (4–6). Currently, renal biopsy remains the “gold standard” for diagnosing, classifying, and assessing the activity of LN (7). Nonetheless, as an invasive procedure, renal biopsy is associated with potential risks, including bleeding and infection, and its invasive nature constrains its utility for the real-time, dynamic monitoring of disease status (8, 9). Furthermore, the inherent subjectivity in pathological interpretation presents significant challenges to the implementation of individualized precision treatment (10). Consequently, the identification of non-invasive and highly sensitive biomarkers that can accurately predict treatment response has emerged as a fundamental necessity for the effective and comprehensive management of LN across the entire disease trajectory.

The scope of biomarkers encompasses a diverse range of biological molecules, including proteins, nucleic acids, metabolites, and immune cell subsets (11). The research focus on LN has transitioned from conventional markers, such as serum complements and anti-double-stranded DNA (anti-dsDNA) antibodies, to innovative biomarkers including urinary monocyte chemoattractant protein-1 (MCP-1) and neutrophil gelatinase-associated lipocalin (NGAL) (12, 13). This shift has further expanded to encompass microRNAs, long non-coding RNAs, and multi-omics profiles (14–16). Particularly in recent years, the explosive development of single-cell sequencing and spatial transcriptomics has provided unprecedented analytical tools for revealing the molecular heterogeneity of LN (17, 18).

Despite the extensive global research on LN biomarkers conducted over the past two decades (2005–2024), there is still a notable deficiency in systematic studies aimed at tracing their developmental trajectory, identifying key research contributors, and predicting emerging research hotspots (19). Bibliometrics, a quantitative analytical method rooted in mathematics and statistics, offers the capability to visually elucidate a field’s collaborative networks, thematic evolution, and foundational knowledge through the use of knowledge map visualization (20, 21).

This study undertakes a comprehensive examination of the literature pertaining to LN biomarkers. Employing bibliometric tools such as VOSviewer, CiteSpace, and Bibliometrix, it systematically analyzes collaboration networks among countries, institutions, and authors, as well as the co-occurrence patterns of keywords and citation burst characteristics, to construct a global research map of this domain. The study aims to elucidate the evolutionary trajectory of research hotspots and the transformations in knowledge structure, with the objective of identifying critical bottlenecks encountered in the transition from biomarker discovery to clinical translation. Additionally, it investigates potential developmental directions within the context of precision medicine, thereby providing a systematic reference for clinical practice and scientific research decision-making.

## 2 Materials and methods

### 2.1 Data collection

This study undertook a systematic literature search utilizing two databases: the Web of Science Core Collection and PubMed. The data retrieval period extended from January 1, 2005, to December 31, 2024, with all searches finalized on November 10, 2025. The search was restricted to publications in English and limited to article-type documents. A comprehensive search strategy is detailed in Supplementary material.

### 2.2 Inclusion and exclusion criteria

**FIGURE 1:**
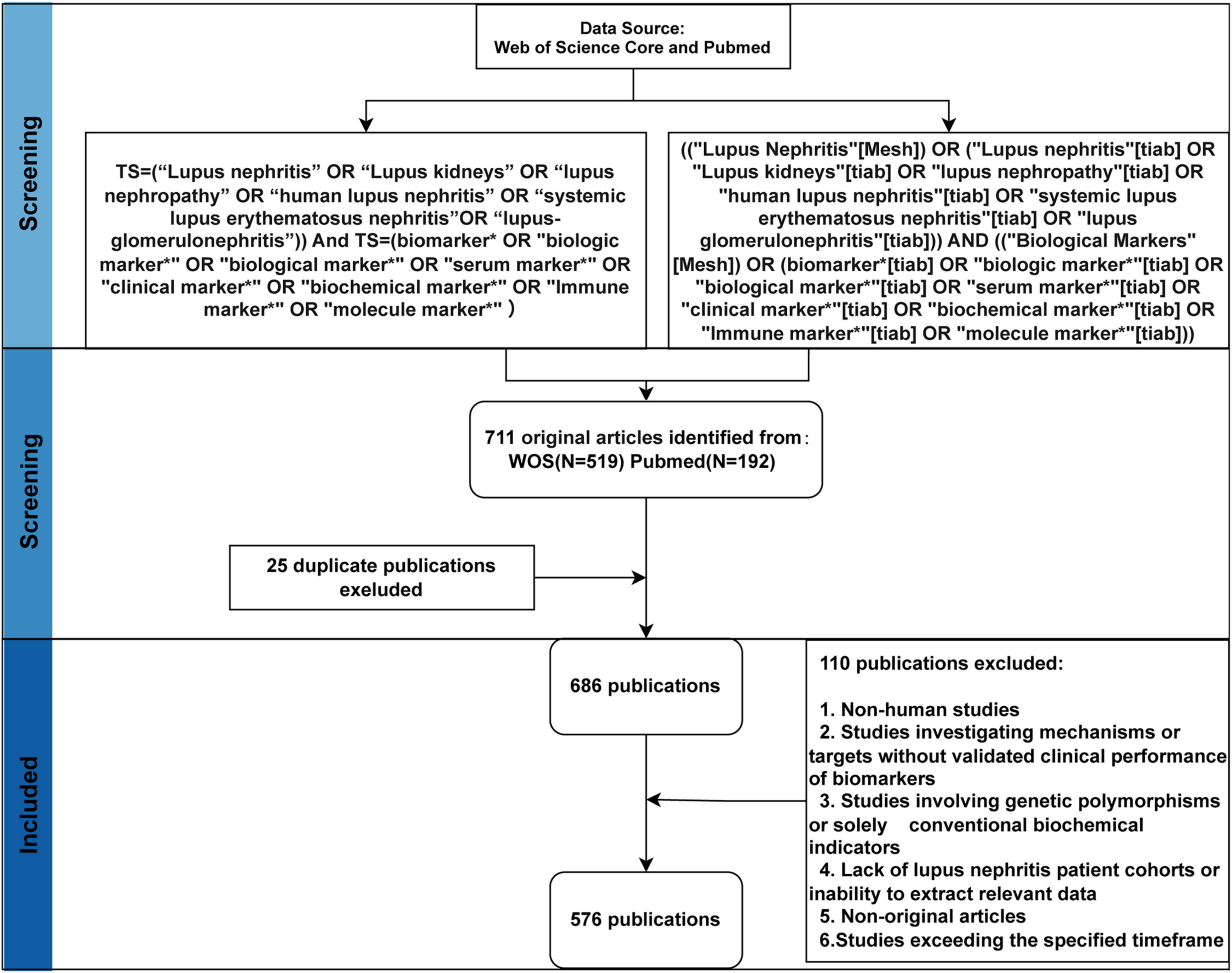
Flowchart for data retrieval and filtering.

This study included literature that met all of the following criteria: (1) English-language original research published between January 1, 2005, and December 31, 2024; (2) Studies involving patients with LN that were pathologically confirmed by renal biopsy, or that met clinical diagnostic criteria for SLE with evidence of renal involvement, or studies comparing groups of LN patients with SLE patients without renal involvement; (3) Original research designs including cohort studies, case-control studies, or cross-sectional studies, providing quantitative evidence of associations between biomarkers (proteins, nucleic acids, metabolites, etc., derived from serum, plasma, urine, renal tissue, or peripheral blood cells) and LN clinical phenotypes; (4) Studies explicitly aimed at investigating biomarker-LN relationships. Literature meeting any of the following criteria was excluded: (1) Non-English publications; (2) Non-human studies (e.g., animal experiments, cell experiments); (3) Studies solely exploring pathogenesis or drug targets without validating the clinical utility of biomarkers; (4) Studies focusing solely on genetic polymorphisms or conventional clinical biochemical indicators (e.g., serum creatinine, estimated glomerular filtration rate, urine protein quantification) without associative analysis with novel biomarkers; (5) Studies that do not include LN patient cohorts or are unable to extract LN-related biomarker data; (6) Non-original research publications (e.g., reviews, editorial materials); (7) Publications dated outside the specified range; (8) Duplicate publications or redundant data; (9) Retraction notices; (10) Irrelevant content or incomplete data. Figure 1 illustrates the flowchart of the literature search and screening process.

### 2.3 Data analysis and visualization

For the literature that met the inclusion criteria, we exported their “Full Record and Cited References” in plain text format. First, we standardized the format and removed duplicates using CiteSpace 6.4.R1. Subsequently, the cleaned data were imported into CiteSpace, VOSviewer, and R-Bibliometrix for further analysis and visualization. In VOSviewer 1.6.20, a synonym dictionary was utilized to merge synonyms, singular/plural forms, and alternative names. Co-occurrence networks were then constructed for countries, institutions, authors, journals, and keywords. CiteSpace 6.4.R1 was employed to identify keywords and references with significant citation bursts, conduct keyword timeline analysis and clustering, and generate journal overlay maps and reference clustering networks. R-Bibliometrix4.3.0 was used to standardize author names and institutional affiliations, as well as to create descriptive statistical charts, including the number of publications categorized by year, country/region, and journal. Further detailed information regarding specific parameters can be found in Supplementary material.

## 3 Results

### 3.1 Publication and citation trends

**FIGURE 2:**
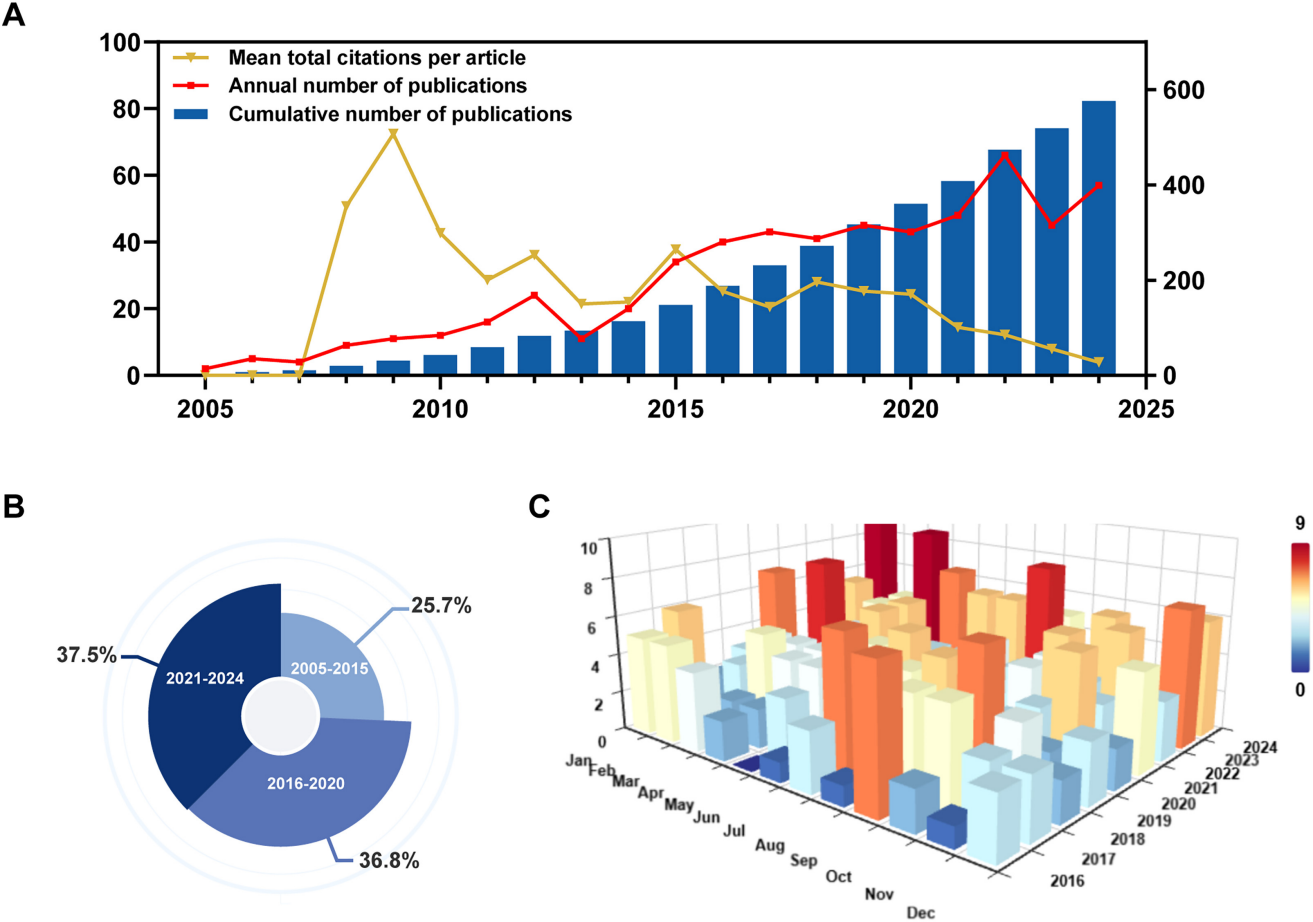
Trends in publications and citations for research on lupus nephritis and biomarkers. **(A)** Trends in annual publications, cumulative publications, and total citations per article. **(B)** The proportion of publications within certain years from 2005 to 2024. **(C)** Monthly publication volume from 2005 to 2024.

This study analyzed 576 literature articles on LN biomarkers published between 2005 and 2024. The annual publication volume in this field has demonstrated sustained growth, categorized into three distinct phases: The period from 2005 to 2010 represented an exploratory phase, during which annual publications consistently remained below 15 articles, culminating in a total of 43 articles. The years 2011 to 2015 marked a phase of stable growth, with annual publications increasing from 16 to 34 articles, resulting in a cumulative total of 95 articles. Since 2016, the field has entered a phase of rapid development, with annual publications consistently exceeding 40 articles. Notably, from 2021 to 2024, cumulative publications reached 216 articles, accounting for 37.5% of the total output. The peak of research activity occurred in 2022, with 66 articles published, indicating a significant increase in research engagement in recent years (Figures 2A,B). From a monthly distribution perspective, publications are generally spread throughout the year rather than concentrated in specific months, indicating sustained research activity and output in this field, which reflects its potential for continued development (Figure 2C).

### 3.2 Countries/regions and institutions

**FIGURE 3:**
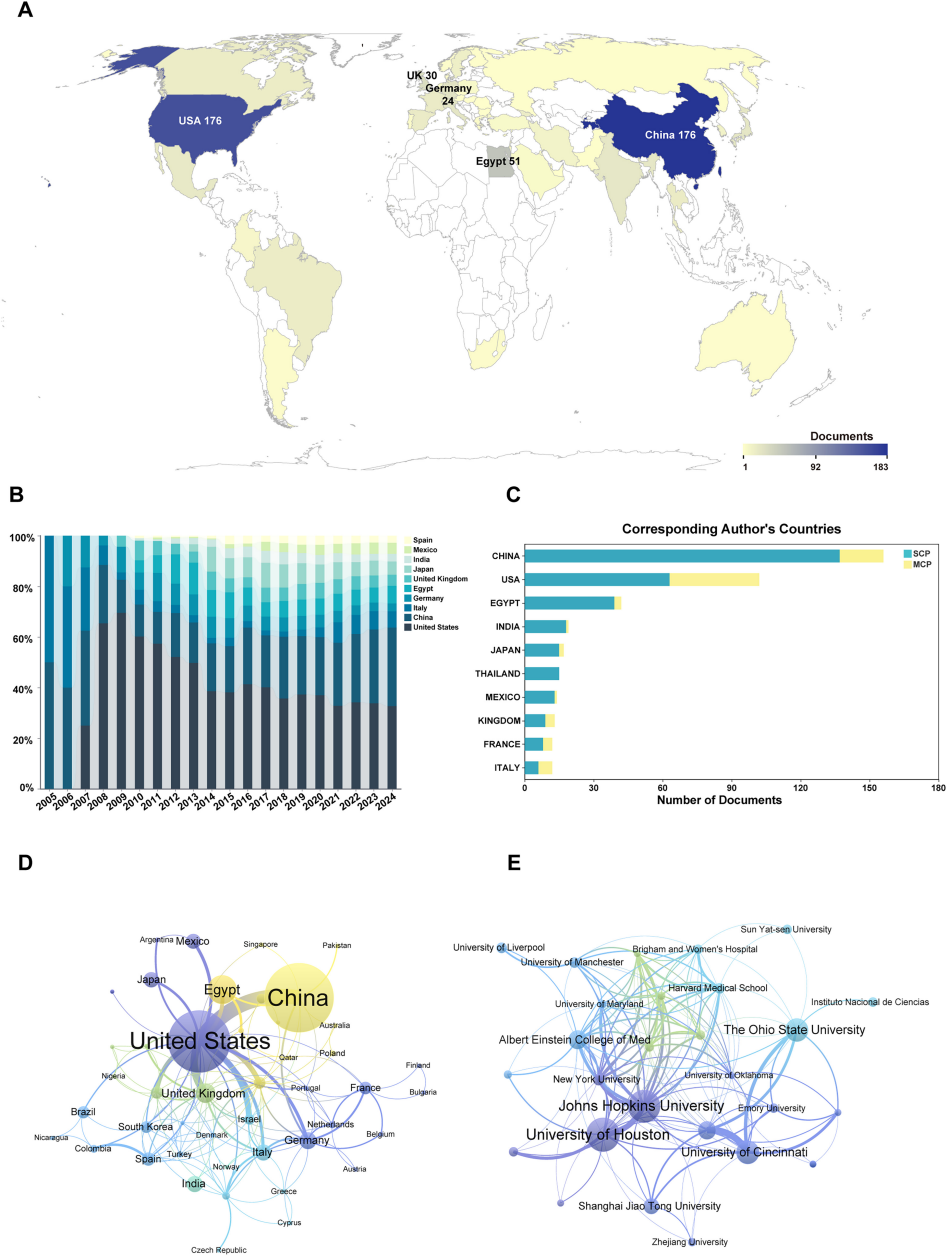
Visualization of countries and institutions in lupus nephritis and biomarker research. **(A)** The global publications’ geographic distribution map. **(B)** Proportion of articles published by countries. **(C)** The top 20 countries/regions of corresponding authors are ranked by the number of publications. **(D)** Cooperation network of different countries in the field of lupus nephritis and biomarkers. **(E)** Collaborative research network for biomarkers in lupus nephritis. MCP: multiple country publications. SCP, single country publications.

This study encompassed 52 countries and regions. China produced the highest number of publications, totaling 176 articles, followed by the United States with 152 articles, Egypt with 51 articles, the United Kingdom with 30 articles, and Germany with 24 articles (Figure 3A). The United States has historically maintained a leading position in LN biomarker research; however, China’s contributions have exhibited a significant upward trend in recent years, establishing it as a major research force (Figure 3B). According to Table 1, the United States had the highest total citations, with 5,333 citations, followed by China with 3,076 citations. In terms of corresponding author publications, China, the United States, and Egypt ranked as the top three contributors (Figure 3C). Among these, China had the highest number of single-country publications (SCP), totaling 137 articles, while the United States led in multiple-country publications (MCP) with 39 articles. Notably, Thailand achieved a 100% SCP ratio. The international collaboration network spanned 44 countries and regions (Figure 3D), with China and the United States demonstrating the most extensive collaboration and maintaining the closest bilateral cooperation.

**TABLE 1:** Top 10 countries/regions and institutions ranked by research volume.

Bank	Country/Region	Documents	Citations	Bank	Institutions	Affiliation	Documents	Citations
1	China	176	3076	1	University of Houston	United States	31	855
2	United States	152	5333	2	Johns Hopkins University	United States	30	1384
3	Egypt	51	701	3	The Ohio State University	United States	21	1205
4	United Kingdom	30	808	4	University of Cincinnati	United States	21	1145
5	Germany	24	804	5	Albert Einstein College of Medicine	United States	17	1077
6	India	22	550	6	Cincinnati Children’s Hospital Medical Center	United States	16	691
7	Italy	22	439	7	Shanghai Jiao Tong University	China	14	432
8	Japan	21	435	8	Peking University	China	12	191
9	Mexico	20	365	9	Cairo University	Egypt	11	141
10	Spain	17	562	10	Nanjing Medical University	China	11	127
							184	

A total of 956 institutions participated in research on LN-related biomarkers. The top ten institutions by publication volume collectively authored 184 articles (Table 1), with the University of Houston ranking first with 31 publications, followed closely by Johns Hopkins University with 30 publications, and both The Ohio State University and the University of Cincinnati contributing 21 publications each. The collaborative network among institutions (Figure 3E) indicates that Johns Hopkins University and the University of Houston maintain the most extensive collaborative relationships, occupying central positions within the network.

### 3.3 Journals and cited journals

This study analyzed a total of 167 journals. Table 2 displays the top 10 journals ranked by publication volume and total citation frequency. Regarding output scale, relevant research was predominantly published in specialized journals within the fields of rheumatology and immunology. Notably, the journal Lupus led with 73 publications, followed by Frontiers in Immunology with 30 publications and Arthritis Research and Therapy with 25 publications. In terms of academic influence, the distribution of journals reveals a distinct hierarchical structure. High-impact journals in the field, such as Kidney International (Impact Factor 12.6, 913 citations) and Arthritis & Rheumatology (Impact Factor 10.9, 1389 citations), played a pivotal role in refining key concepts, constructing theoretical frameworks, and disseminating evidence related to this research topic. Conversely, while Lupus (Impact Factor 1.9, 974 citations) has a relatively low overall impact factor, its strong focus on SLE and LN has facilitated significant knowledge aggregation and citation recirculation. The journal’s high co-citation frequency signifies its pivotal role as a central hub for the accumulation, integration, and dissemination of knowledge in LN research.

**TABLE 2:** Top 10 active and co-cited journals by output and impact.

Journals	Documents (N)	Citations	2024 IF	Co-cited journals	Citations	2024 IF
Lupus	73	1433	1.9/Q3	Arthritis & Rheumatology	1389	10.9/Q1
Frontiers In Immunology	30	482	5.9/Q1	Lupus	974	1.9/Q3
Arthritis Research and Therapy	25	791	4.6/Q1	Kidney International	913	12.6/Q1
Clinical Rheumatology	19	260	2.8/Q2	Journal Of The American Society Of Nephrology	676	9.4/Q1
Plos One	19	766	2.6/Q2	Journal Of Rheumatology	551	3.4/Q2
Rheumatology	17	430	4.4/Q1	Annals Of The Rheumatic Diseases	525	20.6/Q1
Lupus Science and Medicine	15	128	4.0/Q2	Journal Of Immunology	494	3.4/Q2
International Journal Of Rheumatic Diseases	14	168	2.0/Q3	Arthritis Research & Therapy	434	4.6/Q1
Pediatric Nephrology	12	365	2.6/Q1	Nephrology, Dialysis, Transplantation	401	5.6/Q1
Scientific Reports	12	250	3.9/Q1	Rheumatology	376	4.4/Q1

**FIGURE 4:**
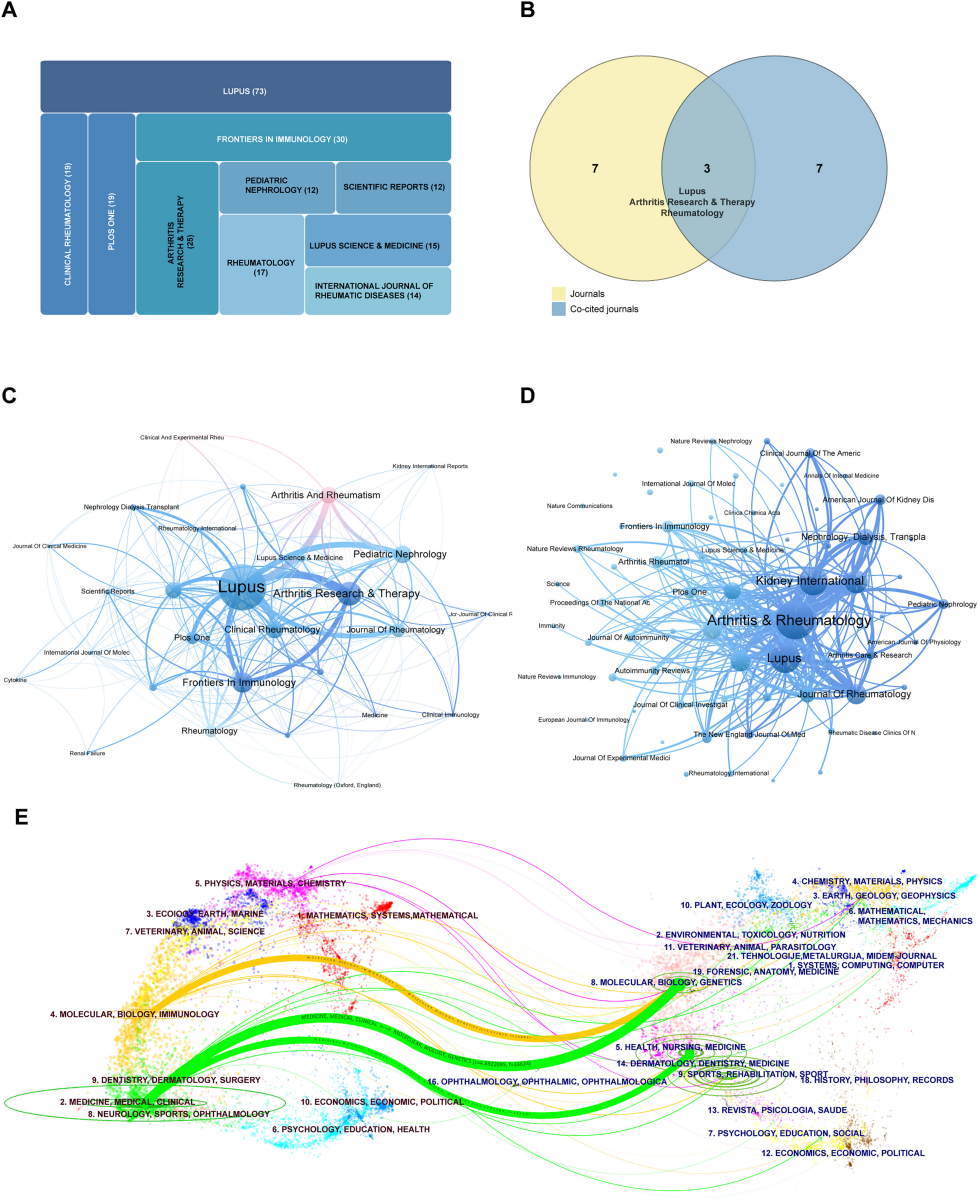
Analysis of journals. **(A)** Top 10 journals in terms of publications. **(B)** Venn diagram of top 10 journals by publication volume and total citations. **(C)** Visualization of journals that have published 5 or more papers. **(D)** Visualization of co-cited journals with at least 40 citations. **(E)** Journal dual stacked chart.

A comparative analysis of the top 10 journals by publication volume and those with the highest co-citation frequencies identified Lupus, Arthritis Research and Therapy, and Rheumatology as common entities in both categories. This overlap underscores their prominence as core journals within the field, fulfilling dual functions in LN research by both disseminating findings and consolidating knowledge (Figures 4A,B). Citation network analysis of the 28 journals with five or more publications (Figure 4C) demonstrates that journals with similar color coding frequently exhibit closer citation relationships. Figure 4D provides a density visualization of journals with citation frequencies of 40 or more, where the intensity of color is directly proportional to citation strength. The journal citation overlay map illustrates distinct patterns of interdisciplinary knowledge flow within this field (Figure 4E), with the left side denoting citing journals and the right side representing cited journals (22). The analysis reveals that research published in journals focused on molecular biology and immunology predominantly references articles from molecular biology and genetics journals (indicated by yellow pathways). This trend underscores the process of translating fundamental discoveries into applications within immunology. In contrast, studies appearing in medicine, medical, and clinical journals concurrently cite research from both molecular biology/genetics and health/nursing/medicine journals (represented by the green pathway). This citation pattern illustrates the dual nature of clinical translational research, which is dedicated to elucidating underlying mechanisms and addressing practical clinical challenges. Furthermore, this pattern confirms that research on LN biomarkers epitomizes an interdisciplinary field driven by clinical needs and supported by fundamental technological advancements.

### 3.4 Authors and co-cited authors

**FIGURE 5:**
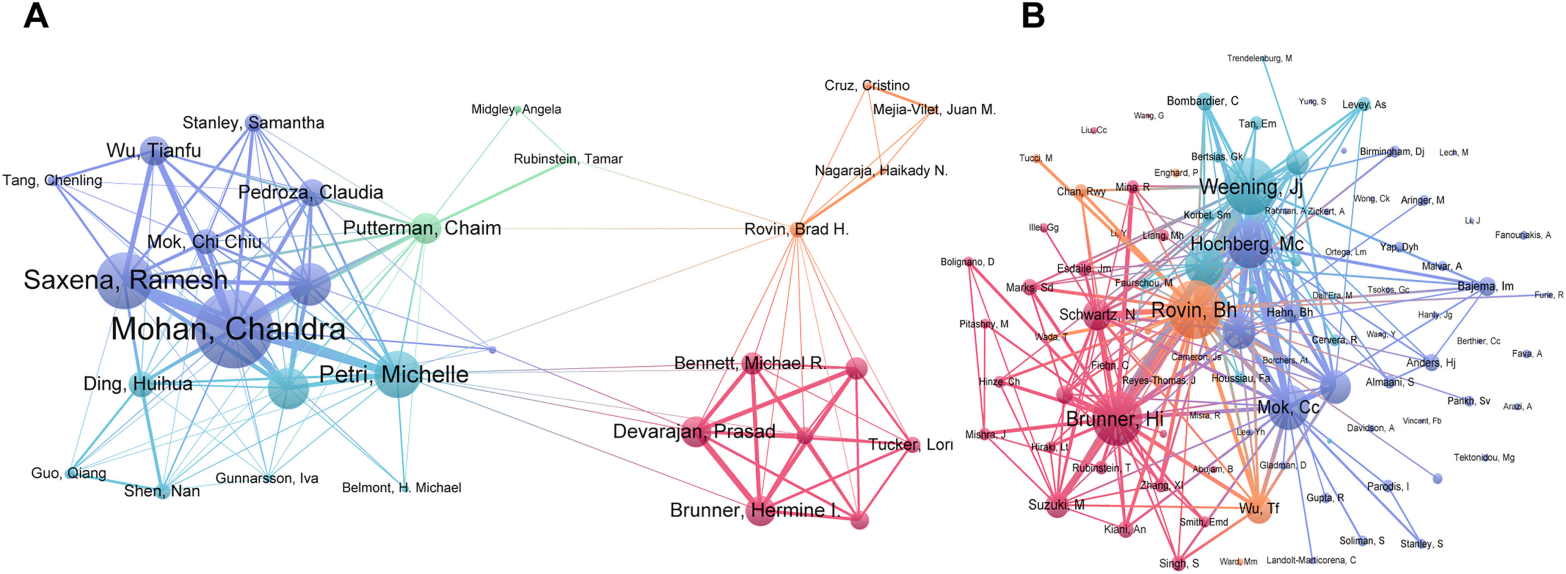
Analysis of authors and co-cited authors. **(A)** Visualization of authors who have published 5 or more papers. **(B)** Visualization of co-cited authors with at least 20 citations.

A total of 3,722 authors have contributed to research in this field. Table 3 presents the top 10 authors ranked by publication volume, whose combined output of 168 papers accounts for 29.17% of the total literature. Among these, Chandra Mohan from the University of Houston leads with the highest number of publications (23), followed by Michelle Petri from Johns Hopkins Medicine (24) and Ramesh Saxena from The University of Texas Southwestern Medical Center (18). Notably, the most frequently cited authors include J.J. Weening, Marc C. Hochberg, Brad H. Rovin, C.C. Mok, and Michelle Petri, each receiving over 140 citations. The author collaboration network analysis (Figure 5A) identified at least 30 core authors (with five or more publications) who have formed tightly-knit yet relatively independent collaborative clusters centered around their respective institutions, such as the University of Houston and Johns Hopkins University, indicating a pronounced institutional cohesion in current high-productivity team collaboration patterns. Additionally, the author’s co-citation network analysis revealed the core knowledge structure of this field (Figure 5B). This network identified 93 authors with citation frequencies of 20 or more, clustered into four major knowledge groups. Among them, J.J. Weening from the University of Amsterdam, cited 211 times, occupies a relatively central position in the network due to his foundational contributions to the revision of LN pathological classification, underscoring the irreplaceable fundamental role of a unified pathological framework in the entire field of biomarker research (24).

**TABLE 3:** Top 10 most prolific authors and most cited authors.

Rank	Authors	Counts	Institution	Co-cited authors	Citations	Institution
1	Chandra Mohan	33	University of Houston	J.J. Weening	211	University of Amsterdam
2	Michelle Petri	23	Johns Hopkins Medicine	Marc C. Hochberg	197	Johns Hopkins University School
3	Ramesh Saxena	18	The University of Texas Southwestern Medical Center	Brad H. Rovin	176	The Ohio State University Wexner Medical Center
4	Brad H. Rovin	15	The Ohio State University Wexner Medical Center	Chi Chiu Mok	148	Tuen Mun Hospital
5	Prasad Devarajan	14	Cincinnati Children’s Hospital Medical Center	Michelle Petri	141	Johns Hopkins Medicine
6	Chaim Putterman	14	Azrieli Faculty of Medicine	Dafna D. Gladman	139	University of Toronto
7	Kamala Vanarsa	14	University of Houston	H A Austin 3rd	138	National Institutes of Health
8	Tianfu Wu	13	University of Houston	Hermine I. Brunner	135	University of Cincinnati
9	Hermine I. Brunner	12	University of Cincinnati	Tianfu Wu	93	University of Houston
10	Ting Zhang	12	University of Manchester	Gabriella Moroni	90	Fondazione IRCCS Ca’ Granda Ospedale Maggiore Policlinico
		168				

### 3.5 Reference analysis

**FIGURE 6:**
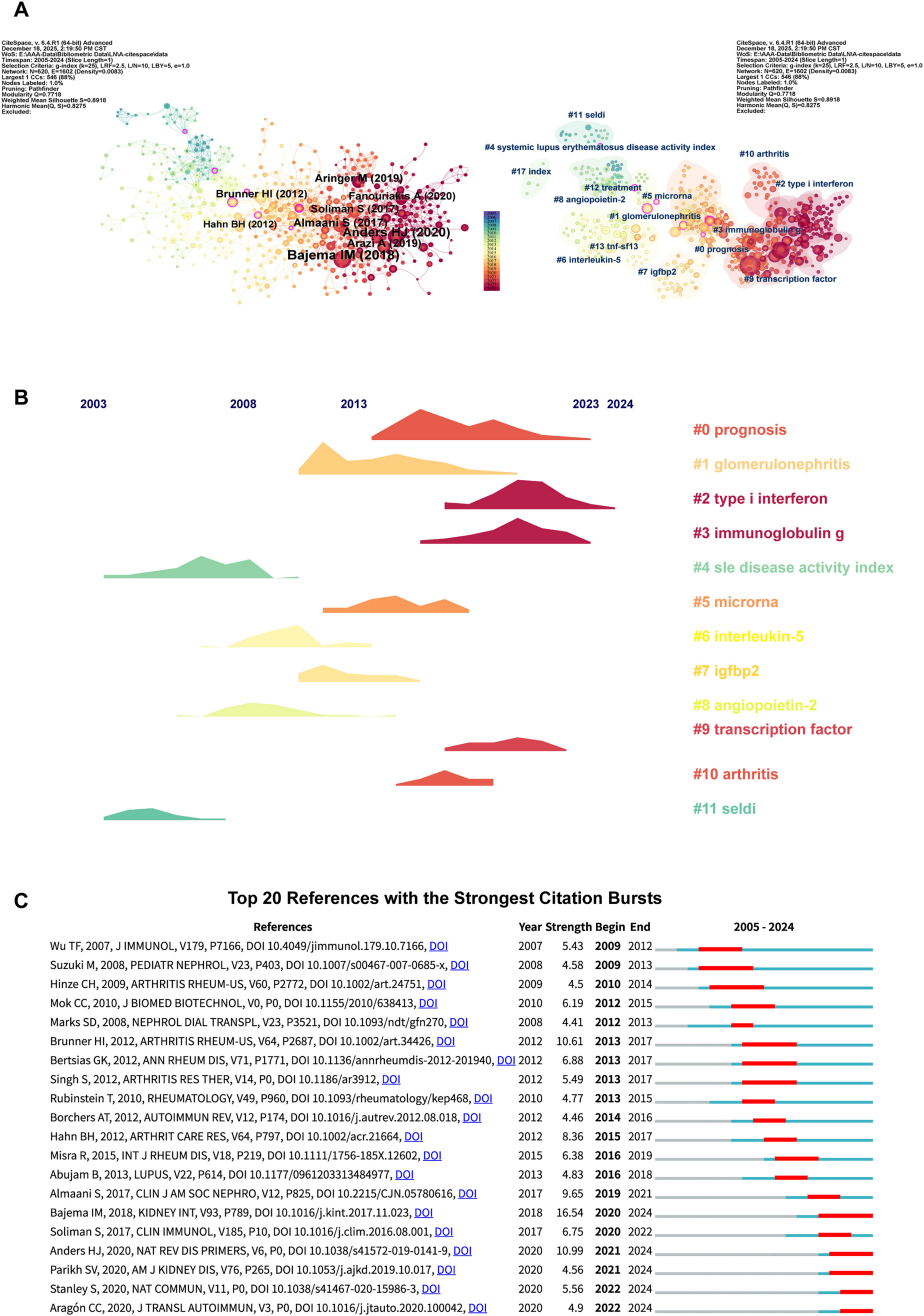
Visualization of co-cited references in the field of lupus nephritis and biomarker research. **(A)** Visualization and cluster analysis of the co-citation references. **(B)** Landscape visualization of the co-citation network. **(C)** Top 20 references for burst strength in lupus nephritis and biomarker research.

The reference analysis elucidates the structural and dynamic evolution of the knowledge base within this domain. An examination of the top 10 most cited articles (Table 4) indicates that the paper titled “Revision of the International Society of Nephrology/Renal Pathology Society classification for lupus nephritis: clarification of definitions, and modified National Institutes of Health activity and chronicity indices,” authored by Bajema et al. and published in Kidney International (Impact Factor = 12.6), occupies the highest rank (Figure 6A). This underscores the international pathological classification standards as the foundational knowledge base and the gold standard for assessment throughout the research field. Furthermore, landscape visualization of the co-citation network (Figure 6B) reveals that early research efforts (2005–2010) predominantly concentrated on biomarker discovery utilizing specific technical platforms, such as SELDI-TOF mass spectrometry, as well as the investigation of classical clinical indices, including the SLE disease activity index, and particular cytokines, such as Interleukin-5 and Angiopoietin-2. With the widespread adoption of high-throughput technologies, research conducted during the mid-period (approximately 2011–2018) has broadened its focus to include comprehensive disease-wide analyses, such as those related to glomerulonephritis, and the identification of novel molecular markers, including microRNA and IGFBP2. Contemporary research frontiers are characterized by an intensified exploration of immune mechanisms, with particular emphasis on specific immune cell types (e.g., macrophages expressing soluble CD163), fundamental signaling pathways (e.g., type I interferon response), and the regulatory roles of immunoglobulins (e.g., immunoglobulin G) and transcription factors. This evolution signifies a transition from studies centered on marker associations to those aimed at elucidating underlying mechanisms. Citation burst analysis indicates that among the top 20 papers with the highest burst intensity, five continue to experience significant citation increases (Figure 6C). These papers likely encapsulate the most recently emerging key concepts, technological advancements, or clinical consensus, representing the most dynamic knowledge units driving current advancements in the field.

**TABLE 4:** Top 20 core references in the research field.

Rank	Cited reference	Total citations
1	Bajema IM, Wilhelmus S, Alpers CE, Bruijn JA, Colvin RB, Cook HT, et al. Revision of the International Society of Nephrology/Renal Pathology Society classification for lupus nephritis: clarification of definitions, and modified National Institutes of Health activity and chronicity indices. Kidney Int. 2018;93(4):789–96.	57
2	Anders HJ, Saxena R, Zhao MH, Parodis I, Salmon JE, Mohan C. Lupus nephritis. Nat Rev Dis Primers. 2020;6(1):7.	45
3	Almaani S, Meara A, Rovin BH. Update on Lupus Nephritis. Clin J Am Soc Nephrol. 2017;12(5):825–35.	30
4	Aringer M, Costenbader K, Daikh D, Brinks R, Mosca M, Ramsey-Goldman R, et al. 2019 European League Against Rheumatism/American College of Rheumatology classification criteria for systemic lupus erythematosus. Ann Rheum Dis. 2019;78(9):1151–9.	25
5	Arazi A, Rao DA, Berthier CC, Davidson A, Liu Y, Hoover PJ, et al. The immune cell landscape in kidneys of patients with lupus nephritis. Nat Immunol. 2019;20(7):902–14.	25
6	Brunner HI, Bennett MR, Mina R, Suzuki M, Petri M, Kiani AN, et al. Association of noninvasively measured renal protein biomarkers with histologic features of lupus nephritis. Arthritis Rheum. 2012;64(8):2687–97.	23
7	Soliman S, Mohan C. Lupus nephritis biomarkers. Clin Immunol. 2017;185:10–20.	22
8	Fanouriakis A, Kostopoulou M, Cheema K, Anders HJ, Aringer M, Bajema I, et al. 2019 Update of the Joint European League Against Rheumatism and European Renal Association-European Dialysis and Transplant Association (EULAR/ERA-EDTA) recommendations for the management of lupus nephritis. Ann Rheum Dis. 2020;79(6):713–23.	22
9	Parikh SV, Almaani S, Brodsky S, Rovin BH. Update on Lupus Nephritis: Core Curriculum 2020. Am J Kidney Dis. 2020;76(2):265–81.	19
10	Stanley S, Vanarsa K, Soliman S, Habazi D, Pedroza C, Gidley G, et al. Comprehensive aptamer-based screening identifies a spectrum of urinary biomarkers of lupus nephritis across ethnicities. Nat Commun. 2020;11(1):2197.	17
11	Malvar A, Pirruccio P, Alberton V, Lococo B, Recalde C, Fazini B, et al. Histologic versus clinical remission in proliferative lupus nephritis. Nephrol Dial Transplant. 2017;32(8):1338–44.	16
12	Hahn BH, McMahon MA, Wilkinson A, Wallace WD, Daikh DI, Fitzgerald JD, et al. American College of Rheumatology guidelines for screening, treatment, and management of lupus nephritis. Arthritis Care Res (Hoboken). 2012;64(6):797–808.	16
13	Bertsias GK, Tektonidou M, Amoura Z, Aringer M, Bajema I, Berden JH, et al. Joint European League Against Rheumatism and European Renal Association-European Dialysis and Transplant Association (EULAR/ERA-EDTA) recommendations for the management of adult and paediatric lupus nephritis. Ann Rheum Dis. 2012;71(11):1771–82.	15
14	Aragón CC, Tafúr RA, Suárez-Avellaneda A, Martínez MT, Salas AL, Tobón GJ. Urinary biomarkers in lupus nephritis. J Transl Autoimmun. 2020;3:100042.	15
15	Der E, Suryawanshi H, Morozov P, Kustagi M, Goilav B, Ranabothu S, et al. Tubular cell and keratinocyte single-cell transcriptomics applied to lupus nephritis reveal type I IFN and fibrosis relevant pathways. Nat Immunol. 2019;20(7):915–27.	14
16	Misra R, Gupta R. Biomarkers in lupus nephritis. Int J Rheum Dis. 2015;18(2):219–32.	14
17	Furie R, Rovin BH, Houssiau F, Malvar A, Teng YKO, Contreras G, et al. Two-Year, Randomized, Controlled Trial of Belimumab in Lupus Nephritis. N Engl J Med. 2020;383(12):1117–28.	13
18	Mejia-Vilet JM, Zhang XL, Cruz C, Cano-Verduzco ML, Shapiro JP, Nagaraja HN, et al. Urinary Soluble CD163: a Novel Noninvasive Biomarker of Activity for Lupus Nephritis. J Am Soc Nephrol. 2020;31(6):1335–47.	13
19	Brunner HI, Bennett MR, Abulaban K, Klein-Gitelman MS, O’Neil KM, Tucker L, et al. Development of a Novel Renal Activity Index of Lupus Nephritis in Children and Young Adults. Arthritis Care Res (Hoboken). 2016;68(7):1003–11.	13
20	Parodis I, Gokaraju S, Zickert A, Vanarsa K, Zhang T, Habazi D, et al. ALCAM and VCAM-1 as urine biomarkers of activity and long-term renal outcome in systemic lupus erythematosus. Rheumatology (Oxford). 2020;59(9):2237–49.	12

### 3.6 Keyword analysis

**FIGURE 7:**
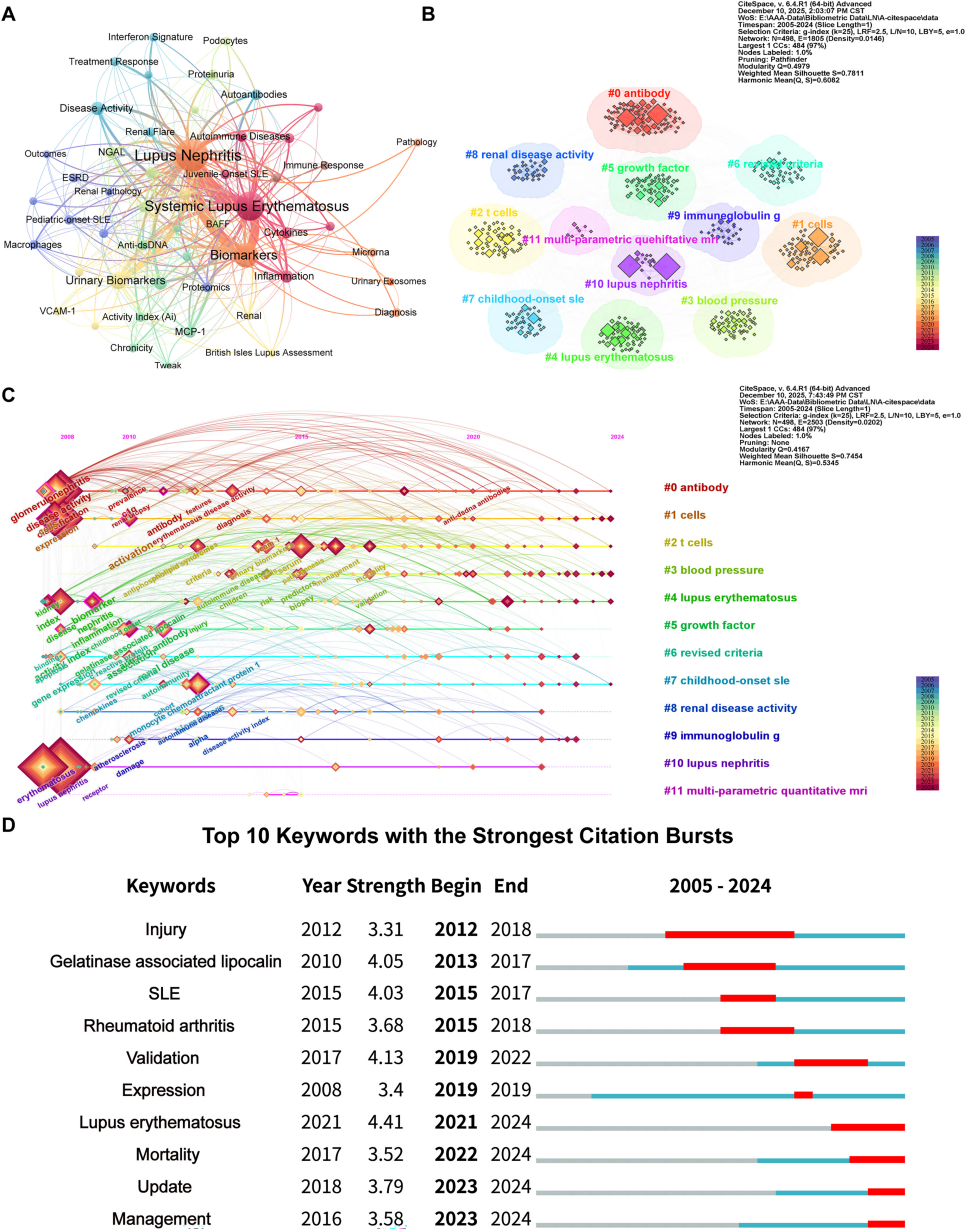
**(A)** The co-occurrence network diagram of keywords related to lupus nephritis and biomarkers. **(B)** Keyword clustering graph of lupus nephritis and biomarkers. **(C)** Timeline visualization graph of lupus nephritis and biomarkers. **(D)** Top 10 keywords for burst strength in lupus nephritis and biomarker research.

Based on the keyword co-occurrence analysis conducted using VOSviewer, a total of 695 keywords were identified, among which 52 keywords appeared with a frequency of at least five occurrences. Table 5 presents the top 20 keywords with the highest frequency. The core terms “lupus nephritis” (396 occurrences) and “systemic lupus erythematosus” (257 occurrences) significantly dominated the frequency metrics. Additional keywords such as “biomarkers” (152 occurrences), “urinary biomarkers” (48 occurrences), “disease activity” (24 occurrences), and “renal biopsy” (24 occurrences) were also frequently observed, outlining preliminary research foci in the field. Clustering analysis of these keywords yielded eight co-occurrence clusters, which can be further summarized into five core research themes (Figure 7A). Group 1 (red cluster) focuses on autoimmune diseases, cytokine/chemokine networks, T cells, and inflammatory responses, with the primary objective of elucidating the immunological basis of LN. Group 2 (green cluster) encompasses disease activity indices, chronicity markers, specific biomarkers (e.g., anti-dsDNA antibodies, MCP-1, TWEAK), and machine learning keywords, reflecting the trend toward integrating multidimensional indicators to construct clinical prediction models. Group 3 (purple cluster) correlates with end-stage renal disease, macrophages, proteomics, and tubulointerstitial lesions, concentrating on the pathological mechanisms and associated markers that drive disease progression toward adverse outcomes. Group 4 (yellow cluster) includes vascular cell adhesion molecules, pediatric lupus, and disease-specific assessment tools, focusing on the mechanisms of vascular endothelial injury and their application in evaluating pediatric LN. Group 5 (blue, orange, cyan, and brown clusters) encompasses a comprehensive theme that broadly covers the complement system, autoantibodies, interferon signatures, podocyte injury, treatment response, and exosomes. This systematically synthesizes the fundamental pathophysiological mechanisms underlying the disease and examines the investigation of biomarkers linked to therapeutic efficacy.

**TABLE 5:** Top 20 most frequent keywords.

Rank	Keyword	Occurrences
1	Lupus nephritis	396
2	Systemic lupus erythematosus	257
3	Biomarkers	152
4	Urinary biomarkers	48
5	Disease activity	24
6	Renal biopsy	24
7	Inflammation	20
8	SLE disease activity index	18
9	MCP-1	16
10	NGAL	16
11	Complement system	15
12	Cytokines	14
13	Autoimmune diseases	13
14	Anti-C1q	12
15	Autoantibodies	12
16	Anti-dsDNA	10
17	Proteinuria	10
18	Proteomics	10
19	T cells	10
20	Activity index	9

Further keyword timeline clustering analysis using CiteSpace (*Q* = 0.4979, *S* = 0.7815) generated 12 major research clusters (Figure 7B). The timeline view reveals a dynamic trajectory of research hotspots: from 2005 to 2010, studies primarily focused on fundamental immune mechanisms such as antibodies and cells; from 2010 to 2020, sustained attention was directed toward pediatric SLE and T cell-related research; since 2020, research emphasis has shown a trend of returning to core immune pathogenesis mechanisms including antibodies, cells, and T cells (Figure 7C). Keyword emergence analysis further reveals that keywords such as “Lupus erythematosus,” “Mortality,” “Update,” and “Management” have shown significant emergence intensity in recent LN research. This signifies a strategic shift in the field’s research focus from biomarker discovery toward optimizing clinical outcomes and disease management practices centered on improving patient long-term survival (Figure 7D).

**FIGURE 8:**
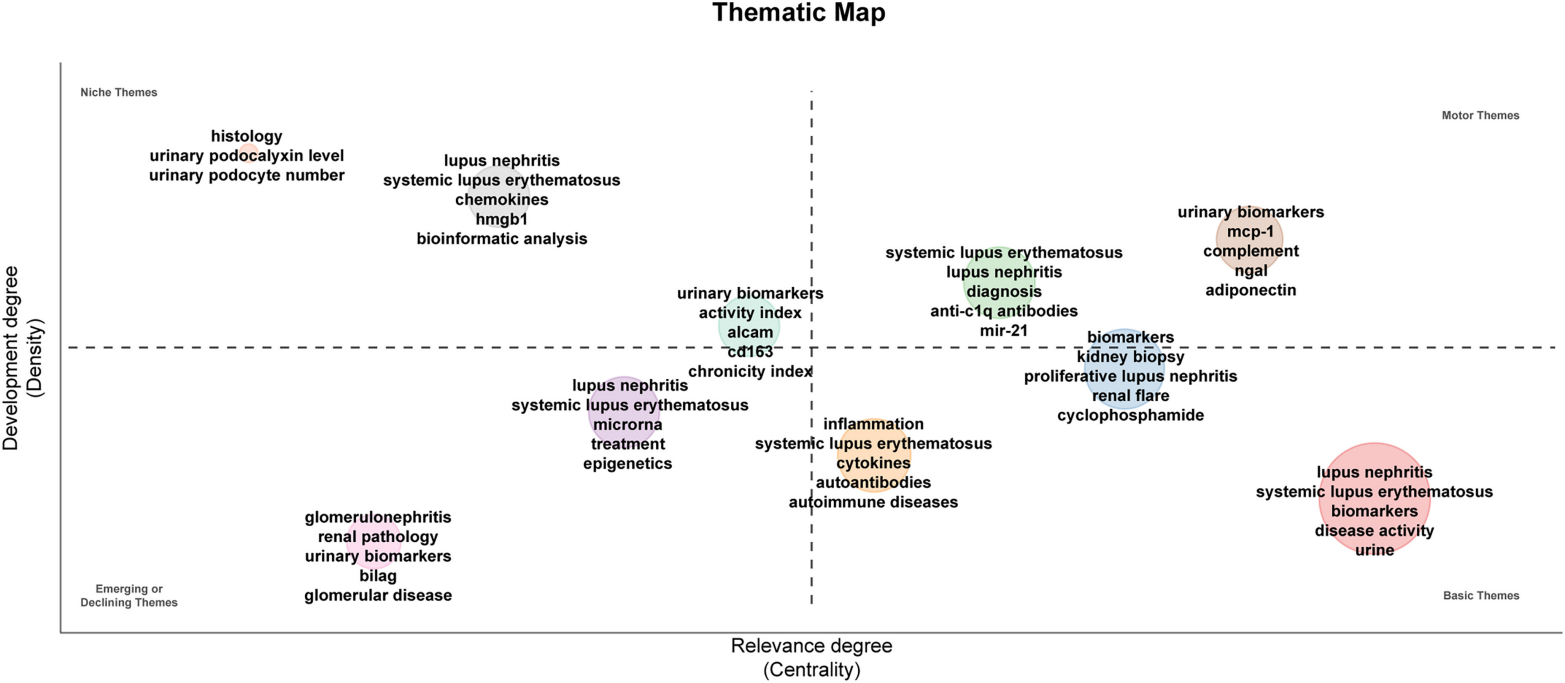
Thematic analysis of lupus nephritis and biomarkers. The horizontal axis shows centrality, while the vertical axis indicates density. Quadrant one features well-established themes, quadrant two contains currently unimportant themes, quadrant three includes emerging or potentially fading themes, and quadrant four covers unimportant foundational themes.

The thematic map comprehensively illustrates the evolutionary trends of research themes (Figure 8). The analysis reveals that the current core thematic network is predominantly focused on LN and the relationship between biomarkers and urinary markers. In contrast, foundational methodological themes, such as “renal biopsy” and “pathological grading,” have become relatively stable over time. Notably, topics like “podocyte injury markers” and “chronicity indices” are transitioning from peripheral to central positions within the map, indicating the field’s ongoing progression towards more pathologically specific and clinically translatable research directions.

## 4 Discussion

### 4.1 General information

This study presents the inaugural comprehensive bibliometric analysis of literature concerning LN biomarkers spanning the years 2005 to 2024. The trajectory of annual publication volume can be delineated into three distinct phases: “initial exploration,” “stable growth,” and “rapid development.” Notably, over one-third of the total publications emerged in the last 3 years (2021–2024), indicating that the field has recently entered a highly active phase. These findings suggest that the field has recently transitioned into a phase of rapid development, a trend likely propelled by the convergence of multiple influencing factors. Since 2016, advancements in technologies such as single-cell transcriptomics, urinary exosome proteomics, and high-throughput protein detection using Olink have matured and become widely adopted, thereby significantly enhancing the accessibility of biomarker discovery and validation studies (25–28). In addition, the 2018 guidance document from the U.S. Food and Drug Administration (FDA), titled “Guidance for Industry: Development of Drugs for Renal Disease,” incorporated “composite renal endpoints combined with biomarkers” into its framework for accelerated approval. This inclusion has objectively increased the demand for translatable biomarkers among clinicians and industry stakeholders (29, 30). Furthermore, the widespread adoption of digital tools, such as electronic data capture (EDC), since the onset of the COVID-19 pandemic, may have reduced the time from implementation to publication for multicenter studies, thereby contributing to the recent increase in research output (31, 32). Additionally, the lack of pronounced seasonal clustering in monthly publication volumes suggests that LN biomarker research is entering a phase of normalization and sustained development.

At the national and regional levels, research on LN biomarkers has established a global research landscape, with China and the United States at its core, alongside contributions from multiple countries. China leads in the number of publications, which reflects a significant increase in investment in this field and the rapid maturation of its research teams. Meanwhile, the United States maintains advantages in total citations (5,333) and citations per paper (35.1), indicating its continued dominance in academic influence. The disparity between “output scale” and “citation impact” may be attributed to differences in research foundations, resource allocation, research type structures (the proportion of basic research versus clinical trials), and the depth of international collaboration between the two countries (23, 33). Further analysis of international collaboration reveals structural disparities in cooperative networks. The United States accounted for 38.2% of MCP, while China, despite leading in SCP, represented only 12.2% of MCP. Some countries, such as Thailand, exhibited 100% SCP characteristics, indicating that their research activities rely more heavily on domestic teams. This suggests persistent structural imbalances in current international collaboration networks, predominantly characterized by a centralized pattern featuring bilateral cooperation between China and the United States. Given that the clinical utility of biomarkers depends on their robustness across diverse genetic backgrounds, the current centralized pattern of collaboration highlights an urgent need for emerging research powers to integrate more deeply into global multicenter networks. The institutional collaboration network displays similar characteristics, with the University of Houston and Johns Hopkins University occupying central positions. However, collaborations remain predominantly concentrated within individual institutions or between closely related academic systems, indicating significant room for expansion in large-scale cross-regional and interdisciplinary cooperation.

At the journal level, the pertinent studies were disseminated across 167 journals, exhibiting notable disciplinary clustering. The publications were predominantly concentrated in journals related to rheumatology, immunology, and nephrology, with “Lupus,” “Frontiers in Immunology,” and “Arthritis Research and Therapy” ranking among the highest in publication volume. This highlights the crucial role of specialized journals in accumulating and disseminating evidence within this domain. However, the influence structure, as revealed by co-citation analysis, does not entirely correspond with publication volume. Authoritative nephrology journals such as “Arthritis and Rheumatology,” “Kidney International,” and the “Journal of the American Society of Nephrology” occupy significant nodes within the co-citation network. This suggests that the field’s frequently adopted key findings and core evidence are primarily refined and disseminated through high-impact core journals. By further integrating publication volume and co-citation performance within the framework of Bradford’s law, it is evident that Lupus, Arthritis Research and Therapy, and Rheumatology represent the core zone of this research theme. However, it is noteworthy that landmark studies are relatively scarce in top-tier general medical journals. This suggests that LN biomarker research is still primarily in the phase of “evidence accumulation” and “candidate validation” rather than “clinical transformation.” The field awaits breakthrough, multicenter randomized controlled trials that can definitively prove the utility of biomarkers in guiding treatment decisions to warrant publication in such high-impact venues.

In an analysis encompassing 3,722 authors, Chandra Mohan from the University of Houston emerged as the most prolific contributor with 33 publications. He was followed by Michelle Petri from Johns Hopkins Hospital, who authored 23 publications, and Ramesh Saxena from UT Southwestern Medical Center, with 18 publications. J.J. Weening from the University of Amsterdam achieved the highest citation count, amassing 211 citations, and his work on revising the pathological classification for LN has become a seminal reference in the field (24). The author collaboration network is characterized by multiple collaborative clusters centered around key institutions; however, the network’s density and inter-cluster communication remain relatively limited. Enhancing collaboration across teams, disciplines, and regions could potentially standardize research methodologies, improve the quality of external validation, and further advance the translatability of biomarkers.

### 4.2 Research hotspots and frontiers

#### 4.2.1 Thematic evolution: from histopathology to mechanistic biomarkers

Keyword citation burst analysis is instrumental in identifying high-impact literature and tracking trends within research fields. Citation peaks in this domain were concentrated between 2009 and 2022, with five publications currently experiencing sustained citation bursts. The persistent demand for standardized frameworks and authoritative consensus continues to underpin the development of the field. The high citation rates of revised pathological classification guidelines and authoritative reviews indicate that, despite rapid innovative iterations, research heavily relies on and continuously consolidates unified pathological gold standards and disease knowledge systems (34, 35). Concurrently, bibliometric analyses reveal that non-invasive methodologies, particularly exemplified by “urinary biomarkers,” have emerged as the most active research trajectory. Cutting-edge high-throughput studies are focused on validating urine biomarker profiles across diverse ethnic populations (36), while concurrently published systematic reviews integrate relevant molecular mechanisms and research advancements (37). Collectively, these studies converge on a central objective: the development of objective, dynamic monitoring tools capable of effectively supplementing or assisting kidney biopsies, thereby advancing the diagnosis and treatment of LN towards precision medicine and minimally invasive strategies.

The keyword thematic analysis and timeline visualization graph effectively illustrate the systematic evolution of research priorities. In recent years, the rapid increase in focus on “biomarker” and “urinary biomarkers,” along with their strong correlation with “mechanisms,” suggests that research is increasingly providing biologically grounded references for optimizing clinical evaluation systems through comprehensive analysis of the renal microenvironment. In this context, while bibliometric maps broadly categorize research into clusters such as “cells” or “biomarkers,” studies at the micro-level are progressively delving deeper into these focal areas. Clusters such as “biomarkers” and “activity index,” as depicted in the Thematic Map, exhibit significant evolutionary potential, indicating that the identification of cell-specific drivers has emerged as a frontier in the field. In alignment with this academic trend, recent studies have concentrated on the detailed characterization of key markers, including CD163+ macrophages and activated neutrophils (38–40). The identification of these molecules elevates biomarkers from mere empirical observations to entities with clear biological logic, thereby serving as a critical bridge between basic research and clinical applications.

#### 4.2.2 Current paradigm: evolutionary trajectory from pathological correlation to dynamic management

Research on urinary biomarkers is systematically transitioning from a focus on “pathological correlation” to “practical clinical application.” This progression is clearly illustrated in the Thematic Map. Although “urinary biomarkers” and “Diagnosis” have developed into distinct and advanced entities within the Motor Themes, their conceptual basis remains firmly anchored in the Basic Themes. This suggests that, at the foundational research level, there is an inherent pathophysiological connection between biomarkers and “Disease Activity.” The dynamic progression of pathological states not only affirms the biological significance of these markers but also forms the underlying rationale for the iterative advancement of diagnostic indicators from theoretical discovery to clinical application. This association, grounded in foundational theory, has been further incorporated into a comprehensive multi-dimensional clinical framework. Stimulated by the increasing frequency of citations for the keyword “management” and the prominence of the “Activity Index” cluster within the “Niche Topics” thematic map, pioneering research in 2025 is realizing three significant strategic advancements. This evolution is characterized by a shift from isolated diagnostic accuracy to real-time monitoring of therapeutic responses, ultimately progressing to long-term prognostic stratification and management. In light of this bibliometric context, we will engage in a thorough discussion across the following three dimensions.

First, regarding diagnostic precision, the liquid biopsy framework in 2025 is achieving a paradigm shift from macroscopic clinical indicators to high-fidelity molecular characterization. By integrating urinary proteomics, exosomes, and exfoliated cells, researchers have successfully constructed a high-fidelity mapping of the intrarenal microenvironment that extends beyond traditional proteinuria assessments. For instance, high-throughput mass spectrometry has identified urinary Afamin as a premier biomarker with exceptional discriminatory power (AUC = 0.99), often rising before clinical symptoms manifest (41). In parallel, the identification of specific acetylated albumin peptides, such as ALB-K36 (AUC = 0.947), allows for the effective differentiation of LN from ANCA-associated glomerulonephritis, even in patients with subclinical proteinuria (42). However, translating these discovery-phase metrics into routine practice necessitates rigorous validation across diverse, multi-ethnic cohorts.

Second, in response to the bibliometric frontier defined by “Management” and the “Activity Index,” research into the real-time monitoring of therapeutic responsiveness is advancing significantly. Neutrophil-derived LAMP1 has emerged as a dynamic surrogate for the renal Activity Index (43). This single-marker approach is complemented by combinatorial strategies, such as the “Active” module of the “5+4” ensemble (integrating CD163, VCAM-1, etc.), and phenotype-specific markers like S100A9, which allow for nuanced mapping of glomerular versus interstitial inflammation (44, 45).

Finally, the quantification of chronicity and long-term prognostic stratification represents the frontier of clinical management. This trend is particularly evident in the investigation of Aquaporins (AQPs) within the context of liquid biopsy. Building upon histological evidence that the downregulation of AQP1/2/3 serves as a sentinel for interstitial fibrosis and tubular atrophy, subsequent studies have successfully translated these findings into non-invasive assays using urinary exfoliated cells or exosomes (46). When integrated with the “Chronic” module of the “5+4” panel—comprising complement components (C5b9), coagulation markers (D-dimer), and extracellular matrix remodeling proteins (Collagen IV and IGFBP-5)—these markers constitute a comprehensive toolkit for identifying irreversible fibrotic remodeling (44, 45).

#### 4.2.3 Future directions: integrated management and multicenter validation

In anticipation of future developments, the emergence of keywords such as “management,” “long-term outcome,” and “risk stratification” indicates a strategic shift from mere diagnostic screening towards a comprehensive prognostic evaluation. Although “Artificial Intelligence” (AI) and “Machine Learning” have not yet established a dominant presence in historical data, the increasing complexity of multi-omics signatures (including urine, serum, and tissue) observed in recent publications suggests their growing importance. The research trajectory indicates that the forthcoming phase will likely emphasize “Integrated Management Models.” Current literature is beginning to propose frameworks that integrate diverse data sources (15).

From a bibliometric perspective, citation metrics are constrained by factors such as publication bias and time lag. Elevated citation rates often reflect the academic dissemination potential of a molecular marker rather than its clinical maturity. Therefore, even if a biomarker serves as a central node within a research network, this primarily indicates its prominence in exploratory research. Substantial challenges remain in translating such biomarkers into routine clinical applications, including low validation study rates, the lack of standardized operating procedures, and inconsistencies across various ethnic cohorts.

Currently, this field continues to face significant translational challenges. Firstly, the majority of biomarker studies remain in the preliminary discovery phase and lack rigorous validation in large-scale, prospective, multi-ethnic clinical cohorts. Secondly, issues related to the reproducibility of candidate biomarkers, combined with the disconnect between laboratory findings and clinical applications, limit their translational potential. Consequently, the indicators previously discussed should be regarded as highly promising “research indicators” rather than fully developed clinical alternatives. The primary challenge moving forward is to bridge this “lab-to-clinical” gap. Research efforts must transition from high-throughput candidate identification to standardized validation within a global, multi-ethnic framework. By addressing bottlenecks such as inadequate sample sizes and inconsistent protocols, the ultimate objective of realizing precision medicine in lupus nephritis can be achieved.

### 4.3 Strengths and limitation

This study represents the first systematic bibliometric analysis in the field of LN biomarkers, aiming to provide comprehensive insights and research directions for investigators and clinicians. However, the study has several limitations: (1) The data sources were confined to WoSCC and PubMed, thereby excluding other databases such as Scopus, which may result in incomplete data coverage and affect the overall comprehensiveness of the findings. (2) Only English-language literature was considered, potentially omitting relevant non-English studies and introducing a language bias. (3) The bibliometric analysis did not include articles published in 2025, which may lead to the omission of influential research outcomes. (4) Bibliometrics utilizes quantitative indicators, including citations and keywords, to evaluate academic impact. However, it is constrained by publication biases and dissemination effects, which hinder its capacity to perform comprehensive qualitative assessments of the methodological rigor, sample representativeness, and reliability of conclusions in original research.

## 5 Conclusion

This study provides a comprehensive analysis of the global knowledge landscape and the evolutionary trajectory of LN biomarker research over the past two decades, utilizing a multidimensional bibliometric approach. The findings highlight a paradigm shift from “experience-driven single-marker discovery” to “mechanism-driven multi-omics systems integration.” Technological advancements, particularly in single-cell sequencing and spatial transcriptomics, have shifted the research focus from peripheral circulatory macro-mapping to in situ analysis of renal local microenvironments. This shift offers theoretical support for noninvasive liquid biopsies that accurately reflect renal pathological phenotypes. Simultaneously, evolving clinical demands are prompting upgrades in research models, with urine biomarker-based multidimensional prediction models enabling noninvasive dynamic assessment of renal activity and chronicity indices. These models have the potential to replace repeated kidney biopsies in certain scenarios. Despite China and the United States leading in research output, significant imbalances persist in international collaboration networks, with large-scale multicenter and multiethnic validation remaining a critical bottleneck for clinical translation.

## Data Availability

The original contributions presented in this study are included in the article/Supplementary material, further inquiries can be directed to the corresponding authors.

## References

[1] Li H Liang J Gao Y Liu M Xia N Kong W et al. IGFBP2 function as a novel biomarker for active lupus nephritis. *J Mol Med.* (2022) 100:1479–91. 10.1007/s00109-022-02241-z 36008635 PMC9470718

[2] Ma H Liu C Shi B Zhang Z Feng R Guo M et al. Mesenchymal stem cells control complement C5 activation by factor H in lupus nephritis. *EBioMedicine.* (2018) 32:21–30. 10.1016/j.ebiom.2018.05.034 29885865 PMC6020800

[3] Ahn SS Yoo J Jung SM Song JJ Park YB Lee SW. Comparison of the clinical implications among five different nutritional indices in patients with lupus nephritis. *Nutrients.* (2019) 11:1456. 10.3390/nu11071456 31252552 PMC6682980

[4] Kronbichler A Bajema I Geetha D Säemann M. Novel aspects in the pathophysiology and diagnosis of glomerular diseases. *Ann Rheum Dis.* (2023) 82:585–93. 10.1136/ard-2022-222495 36535746

[5] Dong C Wu M Liu H Yang K Ma S Guo Y et al. Breaking the cycle: immune complexes, complement activation, and novel immunotherapies in lupus nephritis. *Front Immunol.* (2025) 16:1624850. 10.3389/fimmu.2025.1624850 41142829 PMC12545139

[6] Ding H Lin C Cai J Guo Q Dai M Mohan C et al. Urinary activated leukocyte cell adhesion molecule as a novel biomarker of lupus nephritis histology. *Arthritis Res Ther.* (2020) 22:122. 10.1186/s13075-020-02209-9 32460901 PMC7251704

[7] Bolognesi MM Capitoli G Galimberti S Cattoretti G Bajema I Bruijn JA et al. Dissecting the histological features of lupus nephritis highlights new common patterns of injury in class III/IV. *Ann Rheum Dis.* (2022) 81:1704–11. 10.1136/ard-2022-222620 35940846

[8] Palsson R Short SAP Kibbelaar ZA Amodu A Stillman IE Rennke HG et al. Bleeding complications after percutaneous native kidney biopsy: results from the boston kidney biopsy cohort. *Kidney Int Rep.* (2020) 5:511–8. 10.1016/j.ekir.2020.01.012 32274455 PMC7136322

[9] Li FF Guan YX Li TX Jiang D He ZX Xia P et al. Analysis of hemorrhage upon ultrasound-guided percutaneous renal biopsy in China: a retrospective study. *Int Urol Nephrol.* (2024) 56:1713–20. 10.1007/s11255-023-03860-2 37991602 PMC11001650

[10] Grootscholten C Bajema IM Florquin S Steenbergen EJ Peutz-Kootstra CJ Goldschmeding R et al. Interobserver agreement of scoring of histopathological characteristics and classification of lupus nephritis. *Nephrol Dial Transplant.* (2008) 23:223–30. 10.1093/ndt/gfm555 17981886

[11] Qiu S Cai Y Yao H Lin C Xie Y Tang S et al. Small molecule metabolites: discovery of biomarkers and therapeutic targets. *Signal Transduct Target Ther.* (2023) 8:132. 10.1038/s41392-023-01399-3 36941259 PMC10026263

[12] Guo Q Qiao P Wang J Zhao L Guo Z Li X et al. Investigating the value of urinary biomarkers in relation to lupus nephritis histopathology: present insights and future prospects. *Front Pharmacol.* (2024) 15:1421657. 10.3389/fphar.2024.1421657 39104393 PMC11298450

[13] Barguil Macedo M Wang T Jönsen A Bengtsson AA Gunnarsson I Svenungsson E et al. Neutrophil gelatinase-associated lipocalin (NGAL) in lupus nephritis and beyond. *Lupus Sci Med.* (2025) 12:e001418. 10.1136/lupus-2024-001418 39809521 PMC11751952

[14] Park DJ Joo YB Nam E Lee J Bang SY Lee HS et al. Exploring potential multiple molecular biomarkers that predict treatment response in patients with lupus nephritis. *Sci Rep.* (2024) 14:31422. 10.1038/s41598-024-83057-4 39733104 PMC11682382

[15] Shang S Xia J He G Zheng Y Zhang J Lu H et al. Advances in precision medicine for lupus nephritis: biomarker- and AI-driven diagnosis and treatment response prediction and targeted therapies. *EBioMedicine.* (2025) 117:105785. 10.1016/j.ebiom.2025.105785 40466435 PMC12167120

[16] Mohamed NR El-Fattah ALA Shaker O Sayed GA. The diagnostic and predictive potential of lncRNA CASC2 targeting miR-155 in systemic lupus erythematosus patients with nephritis complication. *Sci Rep.* (2024) 14:30537. 10.1038/s41598-024-81212-5 39690146 PMC11652638

[17] Cui C Cui F Zou Q Zhang Z Jia L. Progress and applications of single-cell RNA sequencing and spatial transcriptome technology in acute kidney injury research. *Mol Ther Nucleic Acids.* (2025) 36:102583. 10.1016/j.omtn.2025.102583 40568028 PMC12192682

[18] Ma M Luo Q Chen L Liu F Yin L Guan B. Novel insights into kidney disease: the scRNA-seq and spatial transcriptomics approaches: a literature review. *BMC Nephrol.* (2025) 26:181. 10.1186/s12882-025-04103-5 40200175 PMC12288332

[19] Drucker E Krapfenbauer K. Pitfalls and limitations in translation from biomarker discovery to clinical utility in predictive and personalised medicine. *EPMA J.* (2013) 4:7. 10.1186/1878-5085-4-7 23442211 PMC3599714

[20] Mohadab ME Bouikhalene B Safi S. Bibliometric method for mapping the state of the art of scientific production in Covid-19. *Chaos Solitons Fractals.* (2020) 139:110052. 10.1016/j.chaos.2020.110052 32834606 PMC7324352

[21] Chen L Ma S Hu D Lin H Zhu Y Chen K et al. Bibliometric study of sodium glucose cotransporter 2 inhibitors in cardiovascular research. *Front Pharmacol.* (2020) 11:561494. 10.3389/fphar.2020.561494 33041801 PMC7522576

[22] Zhao Y Zhu Q Bi C Yuan J Chen Y Hu X. Bibliometric analysis of tumor necrosis factor in post-stroke neuroinflammation from 2003 to 2021. *Front Immunol.* (2022) 13:1040686. 10.3389/fimmu.2022.1040686 36389810 PMC9661963

[23] Ioannidis JPA. Provenance and funding of extremely cited biomedical articles published between 2003 and 2024. *JAMA Health Forum.* (2025) 6:e253045. 10.1001/jamahealthforum.2025.3045 40938617 PMC12432630

[24] Weening JJ D’Agati VD Schwartz MM Seshan SV Alpers CE Appel GB et al. The classification of glomerulonephritis in systemic lupus erythematosus revisited. *Kidney Int.* (2004) 65:521–30. 10.1111/j.1523-1755.2004.00443.x 14717922

[25] Kim J Xu Z Marignani PA. Single-cell RNA sequencing for the identification of early-stage lung cancer biomarkers from circulating blood. *NPJ Genom Med.* (2021) 6:87. 10.1038/s41525-021-00248-y 34654834 PMC8519939

[26] Singh M Tiwari PK Kashyap V Kumar S. Proteomics of extracellular vesicles: recent updates, challenges and limitations. *Proteomes.* (2025) 13:12. 10.3390/proteomes13010012 40137841 PMC11944546

[27] Li N Tang TT Gu M Fu YQ Qian WW Ma NN et al. Single urinary extracellular vesicle proteomics identifies complement receptor CD35 as a biomarker for sepsis-associated acute kidney injury. *Nat Commun.* (2025) 16:6960. 10.1038/s41467-025-62229-4 40730843 PMC12307914

[28] Boluda-Navarro M. Olink(§) explore for high-throughput protein biomarker discovery in cerebrospinal fluid. *Methods Mol Biol.* (2025) 2914:141–63. 10.1007/978-1-0716-4462-1_12 40167917

[29] Zabka TS Lawton M Chu T Friedman GS Peron K Sultana SR et al. Biomarkers of drug-induced kidney injury: use in clinical trials and recent examples of impact on drug development. *Clin Pharmacol Ther.* (2026) 119:608–17. 10.1002/cpt.70134 41277262 PMC12882755

[30] Schuck RN Sekar V. Use of biomarkers in drug development for regulatory purposes. *Clin Transl Sci.* (2025) 18:e70377. 10.1111/cts.70377 41035359 PMC12489174

[31] Nakase H Hayashi Y Hirayama D Matsumoto T Matsuura M Iijima H et al. Interim analysis of a multicenter registry study of COVID-19 patients with inflammatory bowel disease in Japan (J-COSMOS). *J Gastroenterol.* (2022) 57:174–84. 10.1007/s00535-022-01851-1 35089397 PMC8795939

[32] Patruno A Panzarella MO Buckley M Silverman M Salazar E Panchal R et al. Evaluating the impact of electronic health record to electronic data capture technology on workflow efficiency: a site perspective. *JAMIA Open.* (2025) 8:ooaf139. 10.1093/jamiaopen/ooaf139 41180891 PMC12574785

[33] Zhu J Liu W. Comparing like with like: China ranks first in SCI-indexed research articles since 2018. *Scientometrics.* (2020) 124:1691–700. 10.1007/s11192-020-03525-2 32836521 PMC7246301

[34] Bajema IM Wilhelmus S Alpers CE Bruijn JA Colvin RB Cook HT et al. Revision of the international society of nephrology/renal pathology society classification for lupus nephritis: clarification of definitions, and modified national institutes of health activity and chronicity indices. *Kidney Int.* (2018) 93:789–96. 10.1016/j.kint.2017.11.023 29459092

[35] Anders HJ Saxena R Zhao MH Parodis I Salmon JE Mohan C. Lupus nephritis. *Nat Rev Dis Primers.* (2020) 6:7. 10.1038/s41572-019-0141-9 31974366

[36] Stanley S Vanarsa K Soliman S Habazi D Pedroza C Gidley G et al. Comprehensive aptamer-based screening identifies a spectrum of urinary biomarkers of lupus nephritis across ethnicities. *Nat Commun.* (2020) 11:2197. 10.1038/s41467-020-15986-3 32366845 PMC7198599

[37] Aragón CC Tafúr RA Suárez-Avellaneda A Martínez MT Salas AL Tobón GJ. Urinary biomarkers in lupus nephritis. *J Transl Autoimmun.* (2020) 3:100042. 10.1016/j.jtauto.2020.100042 32743523 PMC7388339

[38] Fu Y Wang W Gong N Zheng X Guo X Zhuang K et al. Neutrophil and neutrophil extracellular traps in acute kidney injury: from mechanisms to treatments. *Front Immunol.* (2025) 16:1688207. 10.3389/fimmu.2025.1688207 41169399 PMC12568571

[39] Yao W Chen Y Li Z Ji J You A Jin S et al. Single cell RNA sequencing identifies a unique inflammatory macrophage subset as a druggable target for alleviating acute kidney injury. *Adv Sci.* (2022) 9:e2103675. 10.1002/advs.202103675 35112806 PMC9036000

[40] Liu X Li Z Mao L Lu Y Cheng L Xin X et al. Integration of single-cell RNA sequencing and spatial transcriptomics reveals neutrophil diversity and spatial heterogeneity in acute kidney injury. *Int J Biol Macromol.* (2025) 331:148478. 10.1016/j.ijbiomac.2025.148478 41135905

[41] Lin S Du M Wang J Lai P Yao G Chen W et al. Urine Afamin as a biomarker of lupus nephritis. *Front Immunol.* (2025) 16:1696288. 10.3389/fimmu.2025.1696288 41394867 PMC12698515

[42] Lee YJ Lee EJ Kim K Kim M Yu JY Kim M et al. Urinary acetylated protein as a biomarker of lupus nephritis: a prospective cohort study. *Arthritis Res Ther.* (2025) 27:220. 10.1186/s13075-025-03684-8 41291843 PMC12645779

[43] Ostendorf L Garantziotis P Huang FY Schett G Lederer JA Fava A et al. Neutrophil heterogeneity identifies an association of LAMP1 with proliferative lupus nephritis. *Eur J Immunol.* (2025) 55:e70022. 10.1002/eji.70022 40760840 PMC12322514

[44] Zhang T Castillo J Louis Sam Titus ASC Vanarsa K Sharma V Kureti S et al. Urine proteins reveal distinct coagulation and complement cascades underlying acute versus chronic lupus nephritis. *J Clin Invest.* (2025) 135:e186143. 10.1172/jci186143 41031884 PMC12483563

[45] Hiramoto K Saito S Hanaoka H Kikuchi J Fukui H Hashiguchi A et al. Urinary biomarkers associated with pathogenic pathways reflecting histologic findings in lupus nephritis. *Arthritis Rheumatol.* (2025) 77:298–310. 10.1002/art.43017 39317671 PMC11865699

[46] Melchior M Van Eycken M Nicaise C Duquesne T Longueville L Collin A et al. Decreased expression of aquaporins as a feature of tubular damage in lupus nephritis. *Cells.* (2025) 14:380. 10.3390/cells14050380 40072108 PMC11899336

