# Supplementary material for: Transitioning from invasive to liquid biopsy techniques: a bibliometric analysis and prospective insights on biomarkers in lupus nephritis
Source: Front Med (Lausanne). 2026 May 11;13:1796760. doi: 10.3389/fmed.2026.1796760 (PMC13199017; doi:10.3389/fmed.2026.1796760)
Supplement: Supplementary file 1 [file Data_Sheet_1.docx]

Supplementary Materials

**1. Data collection**

1.1 Search strategy via Web of Science Core Collection

Step:

#1:TS=(“Lupus nephritis” OR “Lupus kidneys” OR “lupus nephropathy” OR “human lupus nephritis” OR “systemic lupus erythematosus nephritis”OR “lupus-glomerulonephritis”))

#2:TS=(biomarker* OR "biologic marker*" OR "biological marker*" OR "serum marker*" OR "clinical marker*" OR "biochemical marker*" OR "Immune marker*" OR "molecule marker*" ）

#3: #1 AND #2

1.2 Search strategy via PubMed

(("Lupus Nephritis"[Mesh]) OR ("Lupus nephritis"[tiab] OR "Lupus kidneys"[tiab] OR "lupus nephropathy"[tiab] OR "human lupus nephritis"[tiab] OR "systemic lupus erythematosus nephritis"[tiab] OR "lupus glomerulonephritis"[tiab])) AND (("Biological Markers"[Mesh]) OR (biomarker*[tiab] OR "biologic marker*"[tiab] OR "biological marker*"[tiab] OR "serum marker*"[tiab] OR "clinical marker*"[tiab] OR "biochemical marker*"[tiab] OR "Immune marker*"[tiab] OR "molecule marker*"[tiab]))

**2. Data analysis and visualization**

2.1 CiteSpace 6.4.R1

The parameter configurations in CiteSpace (version 6.4.R1) were defined as follows:

Time Slicing: A temporal analysis spanning from January 2005 to December 2024, with a duration of one year per slice.

Text Processing: Title, Abstract, Author, Keywords, and Keywords Plus were selected as data sources.

Thresholds: **The top 50 nodes within each temporal slice were identified, and the resulting networks were pruned using the Pathfinder algorithm.**

Clustering: Clustering was performed based on keyword tags (K), and the Log-Likelihood Ratio (LLR) was subsequently employed to extract cluster labels.

Timeline View: The Timeline View was generated by selecting the "Timeline"option based on the keyword co-occurrence clustering analysis.

Burst Detection Configuration: f(x) = ae^-αx^ , α_1_/α_0_: 2.0; α_i_/α_i-1_: 2.0; The number of States: 2; γ[0,1]: 1.0; Minimum Duration: 2.

2.2 R-Bibliometrix 4.3.0

The procedures for retrieving information in R-Bibliometrix (version 4.3.0) were as follows:

The data preparation was conducted using the Biblioshiny web interface for the Bibliometrix R-package. The raw bibliographic records that had been exported from the database were imported for processing, with the author name format being standardized to "Surname and Initials" in order to ensure consistency in author identification and citation analysis. General information was obtained from the "ANALYSIS" menu on the left, and detailed author data were accessed via "Author Profile" under the "Authors" section. Authors with the same surname and initials were distinguished based on their historical publication records and affiliations. To address the issue of author name ambiguity, we performed a secondary verification by cross-referencing institutional affiliations and research fields.

2.3 VOSviewer 1.6.20

Cluster analysis was conducted utilizing the inherent clustering algorithm of VOSviewer, with the resolution parameter configured at 1.0.
